# Government crisis communication innovation and its psychological intervention coupling: Based on an analysis of China’s provincial COVID-19 outbreak updates

**DOI:** 10.3389/fpsyg.2022.1008948

**Published:** 2023-01-26

**Authors:** Shen Zhou, Wensheng Yu, Xinwen Tang, Xiaoqian Li

**Affiliations:** ^1^School of Humanities and Social Sciences, University of Science and Technology of China, Hefei, China; ^2^College of Public Administration, Huazhong University of Science and Technology, Wuhan, China

**Keywords:** outbreak updates, crisis communication, psychological intervention, COVID-19, crisis intervention

## Abstract

Outbreak updates are an integral part of crisis communication during epidemics. Regarding the COVID-19 crisis communication, localities in China present different strategies for managing outbreak updates, which largely determine the effect of crisis communication and the evolution of social psychology. Depending on the analysis of the update texts from 31 provincial (autonomous regions and municipalities directly under the central government) health committees in China, the study found the differences among the provincial outbreak updates and summarizes 12 innovative crisis communication ways. A coupling analysis was applied using the equilibrium-cognitive-psychosocial transition model to further expound on the psychological connotation and intervention potential of the crisis communication innovations. Coupling crisis communication with interventions has a positive effect on designing crisis communication strategies by taking into account psychological factors. It can help construct and optimize the public crisis communication systems and emphasize “care” in modernizing the emergency management capacity.

## 1. Introduction

Outbreak updates are an integral part of crisis communication during epidemics and pandemics. Communicating the epidemic quickly, accurately, and effectively is key to combating the epidemic. In an interview, Yang Gonghuan, former deputy director of the Chinese Center for Disease Control and Prevention (CDC), argued that the core and most profound lesson learned from the severe acute respiratory syndromes (SARS) epidemic was to communicate the epidemic factually and mobilize the whole society toward mass prevention and treatment to overcome the difficulties (Zhao, 2020). *The Prevention and Control of Infectious Diseases Law of the People’s Republic of China (Revised)*, which came into force in 2004 after SARS, clearly stipulates that the state establish a system for public information about infectious diseases. It requires the health administrative departments of the provincial governments to publish information about infectious diseases in their administrative regions regularly to the public when there is an infectious disease outbreak (The National People’s Congress of the People’s Republic of China, 2004).

After the COVID-19 outbreak, localities showed different levels of outbreak update management, which largely determined the effectiveness of crisis communication. The effectiveness of crisis communication also affected the public’s trust in the government and the formation of social panic. What are the differences in the ways the 31 Chinese provinces (autonomous regions and municipalities directly under the central government) communicated about COVID-19? What are the psychological implications of these innovative ways? To answer these two research questions, this study takes the outbreak update texts as an entry point to compare and identify, select innovative communication ways with preservation and institutionalization values from them, and draw lessons based on the coupling of psychological interventions that have the potential to benefit all citizens.

The rest of the article is organized as follows. The second part describes the research background, which retraces the relevant studies on crisis communication as a means of crisis psychological intervention during COVID-19; the third part describes the research design, extracts a number of features in terms of both content and form, presents codes based on the practical characteristics of the outbreak updates from each province, and determines whether these innovations have the potential for intervention at the country-level scale. Five binary coupling questions are raised within the corresponding five elements of the three basic models of crisis psychological intervention. The three basic crisis intervention models are the equilibrium model, the cognitive model, and the psychological transition model, which describe how the intervention mechanism affects individuals when they are psychologically imbalanced, are recognizable, and do influential errors, respectively; in the fourth part, we analyze the coding results and summarize 12 innovative ways for communicating epidemic situations; the fifth part is the coupling analysis by matching innovative ways, according to their possible potential for large scale intervention. The Delphi method is used to carry out the study on the coupling. The experts were given a 5 × 12 table (five coupling questions and 12 innovative ways) and asked to tick the cells where the coupling was considered. Finally, the discussion and conclusion summarize the whole study and, from the perspectives of communication and psychology, propose crisis communication strategies that better balance information communication and psychosocial intervention. The present research expands the theoretical work of crisis communication for intervention, leads to further practice progress in leveraging daily-updated communication for nationwide and even worldwide intervention, provides important variables and paves the way for future empirical effect research, and promotes emphasizing “care” in the modernization of emergency management system.

## 2. Research background

From crisis communication to crisis intervention, the essence is to intervene in the public’s mental health by using innovative ways of crisis communication so as to achieve the purpose of crisis management.

The current research frameworks of COVID-19 crisis communication are mainly based on the perspective of management. Malecki et al. (2021) used the hazard and outrage framework to analyze the risks of the current pandemic and provide crisis communication strategies for professionals. Zhang and Ran (2020) viewed crisis risk communication of COVID-19 from the perspective of “social amplification of risk.” From the field theory perspective, Hou and Du (2020) took the entry, transition, and exit performance of the Guangdong Provincial Information Office in the epidemic prevention and control press conference as an example to analyze the innovative government news release mechanism for major public health events.

As for crisis intervention, Lindemann (1944) first proposed a basic crisis intervention theory that emphasizes that a person should not be overly immersed in pain such as the loss of a loved one, and he believes that grief is normal and temporary and can be treated by short-term crisis intervention; Psychologist Caplan (1964) built on his predecessors and extended the application of crisis intervention to all developmental and situational events. Current COVID-19 crisis interventions focus on specific groups. Some investigated the impact of an online intervention program on anxiety and depression levels, as well as physical symptoms, in frontline nurses fighting the COVID-19 pandemic to provide an empirical basis for crisis psychological interventions for frontline medical staff (He et al., 2022). Some found that parents, especially mothers, experienced more psychological stress during the outbreak, which may have had an impact on their children’s mental health, while adolescents with higher levels of self-compassion were less psychologically affected by their mothers’ anxiety (Zhou et al., 2022).

Researchers are recently paying more attention to using crisis communication for crisis intervention in the COVID-19 setting: Ribeiro et al. (2021) summarized the Portuguese’s telephone-based psychological crisis intervention, which can effectively provide brief, appropriate, and timely psychological help. Lu et al. (2022) explored the changes in public sentiment in China at different stages of the COVID-19 outbreak, established an online work platform, and constructed a new model of online intervention of public emotions during the epidemic, which can effectively regulate the negative emotions of the public and provide a reference for the government’s emergency management system. Qin et al. (2020) introduced the institutional settings, the management systems, treatment methods, information communication systems, and other interventions in the United States, Israel, and other developed countries to deal with psychological crises of public health emergencies. Havsteen-Franklin et al. (2020) conducted a systematic review to establish the effectiveness of art-based interventions.

However, previous research in this field has failed to (1) systematically explore the psychological connotation of COVID-19 crisis communication; (2) look at the problem at the citizen scale; they currently focus on specific subgroups, such as frontline nurses, parents, and pediatric health workers; and (3) conduct research in a Chinese context. Most of the established studies on crisis communication in China used data from Weibo and WeChat and did not use data from the official health commission website to examine authoritative crisis communication.

## 3. Research design

### 3.1. China’s central–provincial relations in crisis communication

China’s administrative system is characterized by complex central–provincial relations. In the health sphere, the National Health Commission of the People’s Republic of China represents the center and is in-charge of organizing the formulation of national health policies for the development of health care and overseeing their implementation. The health commission of a province (the present research studied the 31 provincial health committees in China) implements the center’s guidelines, policies, and regulations with some flexible room according to the local’s specific situation. Therefore, some significant local differences are reflected in COVID-19 prevention and control.

**FIGURE 1:**
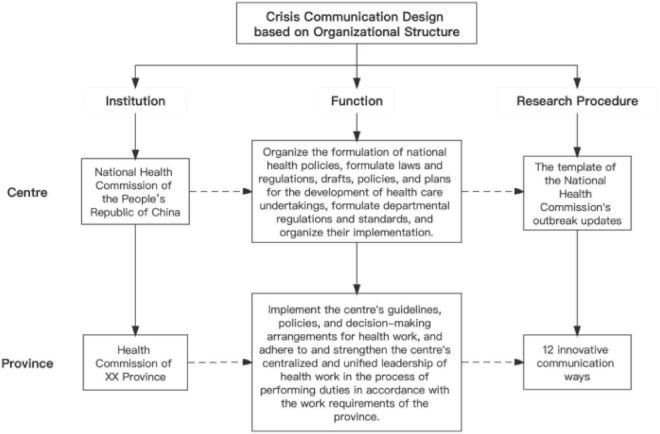
China’s central–provincial relations in crisis communication.

Regarding COVID-19 crisis communication, the study organizes the guidance from the central and localities’ innovative methods as follows (see Figure 1).

### 3.2. Research process

**FIGURE 2:**
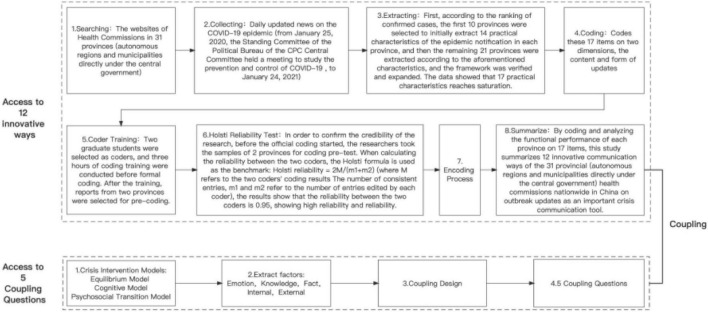
Research process.

The research process of the study (e.g., from initial searching to collecting, comparing and extracting, initial coding, training, reliability testing, and encoding and coupling) is demonstrated in Figure 2.

### 3.3. Identification of crisis communication innovation

The websites of health commissions at all levels in China are the primary platform for the government to communicate epidemic information. It is also the primary way for the public to obtain official information. In this study, provincial health commissions’ outbreak updates were used as the research object. By combing the situation of provincial outbreak updates, we found that the provinces adopted the template of the National Health Commission’s outbreak updates. The template related to the basic data information of the epidemic was described, mainly the number of new confirmed cases, cumulative confirmed cases, suspected cases, new asymptomatic infections, and the number of close contacts traced, etc. This updated template was also used as the baseline condition for this study.

*Template: From 0 to 24 o’clock on a specific date in 2020, the present province reported a certain number of new confirmed cases of Covid-19 and a certain number of cases from abroad. Among them, how many cases in what city, how many cases in what city…… As of midnight on a certain date, how many confirmed cases of Covid-19 have been reported in the province, how many cases have been cured and discharged, and how many have died of the disease. How many cases are still under hospital treatment, of which: how many cases are severe, how many cases are critical, how many cases are common types…… how many existing suspected cases,…… how many new cases of asymptomatic infection in the province,…… how many close contacts have been traced so far and how many have been discharged.……*

Building on the template baseline, we extracted 17 items (practice characteristics) of each province’s outbreak updates. The study codes these 17 items based on two dimensions, the content and form of updates, which form the basis of the analysis for this study (see Table 1).

**TABLE 1:** Coding items.

	Code item (yes = 1; no = 0)
Content of the updates	Is there a description of the case’s attendance at the hospital?
	Is there a description of the case’s life trajectory?
	Are there demographics on the overall cases?
	Is there a description of the circumstances of the fatal cases?
	Is there a reported cure rate?
	Are there reports of re-positive cases?
	Are there any friendly tips/expert advice?
	Is there information on regional risk levels?
	Are there updates on case discharge information?
	Is the receipt/use of the donation published?
Form of the updates	Are multiple briefings conducted daily?
	Is multilingualism used for communication?
	Are charts used for the briefing?
	Is synthesized speech used for communicating?
	Are outbreak map visualization used for updates?
	Are trend charts used to inform outbreak development?
	Are press conferences held for outbreak information releases?

### 3.4. Crisis intervention models and coupling design

James and Gilliland (2016) suggested three theoretical models of crisis intervention: the equilibrium model, the cognitive model, and the psychosocial transition model. The equilibrium model assumes that individuals in crisis are usually in a state of psychoemotional imbalance, and their existing coping mechanisms cannot solve their problems. In the equilibrium model, crisis intervention works by stabilizing individuals to restore them to their precrisis state of equilibrium. The cognitive model suggests that the psychological damage caused by a crisis is mainly attributed to the individual’s misjudgment of the crisis event and the situation surrounding the event, correcting the irrational and self-defeating components of the individual’s cognition and providing professional knowledge and objective information so that the individual can regain control over the life crisis. The psychosocial transition model considers that the factors affecting the individual’s state of crisis can be divided into internal and external components: internal qualities such as the psychological resources and coping abilities that the individual possesses and the external environment consisting of peers, family, occupation, religion, and community. The purpose of crisis intervention is to combine appropriate coping styles within individuals with social support and environmental resources so that the victim has more problem-solving options.

The present study applies the three crisis intervention models and proposes corresponding coupling questions for communication as intervention analysis (see Table 2). The Delphi method is used to carry out the study on the coupling. Each of the 15 experts in communication and psychology studies at the University of Science and Technology of China was given a 5 × 12 table (five coupling questions and 12 innovative ways) and asked to tick the cells where the coupling was considered. The coupling is established when more than eight experts check the cell on a particular item.

**TABLE 2:** Models, factors, and coupling questions.

Models	Factors	Coupling questions
Equilibrium Model	Emotion	Can this crisis communication way directly work on alleviating negative public stress?
Cognitive model	Knowledge	Can this crisis communication way enhance public knowledge that helps the prevention and control of the pandemic?
	Fact	Can this crisis communication way help the public correctly perceive the objective *status quo*?
Psychosocial transition model	Internal	Can this crisis communication way provide psychological resources and enhance the coping ability of the public, especially for vulnerable groups?
	External	Can this crisis communication way help the public to obtain information related to social support and environmental resources?

## 4. Innovative ways

Overall, the study found that all provinces used the updated template for the baseline condition in their updates. In the following, we report the coding results in content and form, respectively.

### 4.1. Content of the updates

First, approximately 23% of provinces (*n* = 7) notified the attendance of cases. For example, the Shaanxi Provincial Health Commission notified the attendance of confirmed cases on the same day of its daily outbreak updates, as shown in the following example (4 February 2020).

*“Patient 1, male, 65 years old, currently residing in Danfeng County, Shangluo City. He arrived in Danfeng County on January 21 from Utopia County, Hubei Province. Developed symptoms on the 26th and visited Xiaohe Health Office in Taoyuan Village, Shang Town, and Danfeng County on the same day. Symptoms worsened on February 2, and Danfeng County Hospital visited the same day. Diagnosed with Covid-19 on February 4. He is currently in quarantine at Shangluo City Central Hospital and is in stable condition.”*

Second, approximately 10% of the provinces (*n* = 3) notified the life trajectory of the cases. For example, the Shaanxi Provincial Health Commission has notified the life trajectory of the confirmed cases from the previous day since 7 February 2020, as shown in the following example (15 February 2020).

*“Case 1: Female, 63 years old, currently residing in Gumeng East District, Beijie Street Office, Jincheng City.*

*On January 20, 2020, from 8:00 to 9:00, She was shopping near the Golden Shield Market on North Street, and from 9:00 to 12:00, She was at the Temple River Jiayuan on North Zechu Road. At 17:00, She was walking in the small area where he lived, and at 17:30, She accompanied his family to the second floor of the Urban Chinese Hospital for medical treatment. At about 18:00, She was driven by her family to the emergency department of Jincheng City People’s Hospital, where she accompanied her family for medical treatment.*

*On January 21, 2020, her family drove her home from Jincheng City People’s Hospital at about 01:00. She took bus No. 2 to accompany her family again to Jincheng City People’s Hospital between 09:00 and 12:00. She was then sent home by his family.*

*On January 23, 2020, at about 14:00, she walked to the second floor of Zhejiang Trade City and returned home about an hour later.*

*January 24 - February 13, 2020, Occasional walks in her living area.*

*On February 6, 2020, she felt unwell, felt weak, anorexic, feverish, and took medication at home on his own.*

*On February 14, 2020, she attended the outpatient clinic of the Urban Chinese Hospital at around 09:00 and took a temperature of 37.2 degrees. She attended the fever clinic of the Ancient Shuyuan Mining Hospital at 14:30 and was taken by her family to the Jincheng University Hospital for consultation and hospitalization at around 17:00.*

*The diagnosis was confirmed on February 15, 2020.”*

Third, approximately 16% of the provinces (*n* = 5) reported on the demographics of overall cases. For example, the Guangdong Provincial Health Commission reported the daily statistics on the gender and age of cumulative confirmed cases, as shown in the following example (27 February 2020).

*“662 males and 685 females between 2 months and 90 years.”*

Fourth, approximately 13% of provinces (*n* = 4) reported fatal cases. For example, the Chongqing Municipal Health Commission informed about the fatal cases in a routine press briefing as shown in the following example (3 February 2020).

*“Patient Lai, male, 51 years old, from Wanzhou district, was diagnosed with Covid-19 on January 24. Examination test results showed that the patient had several underlying diseases: type 2 diabetes mellitus, old tuberculosis, hepatitis B, fatty liver, poor cardiac, pulmonary, and hepatic function, high specific indexes of myocardial infarction, interstitial changes in both lungs with extensive exudation, and insufficient oxygenation capacity. After admission, though via several municipal and district-level expert consultations, clinical symptomatic active treatment, the shortness of breath, dyspnea, and other symptoms gradually aggravated. At 12:30 on February 2, 2020, the patient’s heart rate suddenly dropped, and oxygen saturation dropped sharply to 38%, to the municipal and Wanzhou area expert group all-out rescue. However, the patient’s heart rate never recovered. At 13:44, the case passed away.”*

Fifth, a few provinces (Beijing, Anhui) informed about the cure rate. For example, Beijing had reported the cure rate since 16 March 2020 as shown in the following example (20 March 2020):

*“As of 24:00 on March 19, a cumulative total of 415 confirmed local cases were reported, with 378 cases cured and discharged, a cure and discharge rate of 91.1%.”*

Sixth, only two provinces (Tianjin and Hainan) notified the local repositive cases. For example, the updates from the Tianjin Municipal Health Commission read as follows (29 February 2020).

*“The 29th and 43rd confirmed cases in Tianjin have been transferred to the Haihe Hospital for observation and treatment after being discharged from the hospital and retested positive for nucleic acid during their continued intensive quarantine and observation. The 29th case has been under medical observation at the centralized isolation and observation site in Heping District since its discharge on February 13; the 43rd confirmed case, after its discharge on February 11, has been under medical observation at the centralized isolation and observation site in Baodi District. On February 27, the two retested positive for nucleic acid and were again transferred to Haihe Hospital.”*

Seventh, approximately 26% of the provinces (*n* = 8) provided warm tips or expert advice on outbreak updates. For example, the Anhui Provincial Health Commission added information such as “warm tips” or “brief analysis of the outbreak” at the end of the daily outbreak information, as shown in the following example (25/26 February 2020).

*“Warm Tip: CDC experts reminded that the general public should still insist on wearing masks, washing hands regularly, and not gathering.”*

*“Brief analysis of the epidemic: the current momentum of the spread of the epidemic in our province has been initially contained, and the positive and positive trend continues to expand. As of 24:00 on February 24, the province’s total number of confirmed cases is 989, with zero new confirmed cases reported for three consecutive days; 692 cases were cured and discharged, 35 cases are expected to be discharged today, and the cure rate will reach 73.5%; the number of critically ill patients dropped from a peak of 16 cases to 0 cases. The province continues to show the stage of “four no’s”: no new confirmed cases, no new suspected cases, no critically ill patients, and no new deaths.”*

Eighth, approximately 16% of the provinces (*n* = 5) regularly published a categorized list of epidemic risk levels or a zonal grading scale on a district basis. The risk levels of counties and districts are assessed and dynamically adjusted as high, medium, or low risk according to the changes in the epidemic situation. For example, the list of epidemic risk levels published by the Jiangsu Provincial Health Commission reads the following example (29 February 2020).

*“As of 24:00 on February 27, 2020, counties (cities and districts) (i) Low-risk areas (n* = *59) Nanjing: Lihe District, Lishui District, Gaochun District; Wuxi: Xishan District, Yixing City. (ii) Medium-risk areas (n* = *8) Wuxi: Huishan District, Jiangyin City; Xuzhou. Suining County. (iii) High-risk areas (n* = *1) Huai’an City: Huai’an District.”*

Ninth, two provinces (Shanghai and Chongqing) broadcasted cases of healing and discharges separately, informing the number and the cumulative number of discharges on the same day. For example, the Chongqing Health Commission communicates hospital discharges with “Good News!” as in the following example (15 March 2020).

*“Good news! A confirmed case in Chongqing Changshou District was cured and discharged from the hospital. The designated hospital and medical staff meticulously treated and cared for the patient. The expert group assessed that the patient met the latest National Health Commission’s discharge criteria for confirmed cases of Covid-19, and he was discharged on March 15. A total of 570 patients suffered Covid-19 have been cured and discharged from the city.”*

Tenth, two provinces (Shandong and Yunnan) made public announcements on the receipt of donations, public announcements on the distribution of medical protection materials by the Material Security Group, and announcements on the allocation of charitable donations, and the use of donations. For example, the announcement of Shandong Province on the donation to Huanggang City, Hubei Province, reads the following example (25 March 2020).

*“It was decided to allocate 231,658,700 yuan from the epidemic prevention and control fundraising pooling account for the purchase of key medical equipment for epidemic prevention and control, the construction and operation and maintenance of important medical facilities, and other expenditures related to epidemic prevention and control in Huanggang City, Hubei Province, and the above funds have been allocated. Another RMB 1,177,300,000 was allocated for the procurement of 66 sets of teleconferencing and diagnosis and treatment system equipment by the Shandong Provincial Health Commission to support Huanggang City in Hubei Province and the designated hospitals in the five counties and cities, all of which have been delivered on February 22, 2020.”*

### 4.2. Form of the updates

First, approximately 26% of the provinces (*n* = 8) notified about the outbreak multiple times a day (two or more times). For example, Shanghai notified two times a day from 19 February to 17 March 2020. Municipalities reported two times a day. As the pressure to combat the epidemic eased, the update frequency returned to one time a day in all provinces.

Second, approximately 16% of the provinces (*n* = 5) communicated the epidemic in multiple languages. For example, Hunan Province communicated information about the outbreak in seven languages, Chinese, English, French, German, Japanese, Korean, Lao, and Russian. The Foreign Affairs Office of the provincial government is responsible for translating the epidemic situation into other languages and publishing it on the official website of the Provincial Health Commission.

**FIGURE 3:**
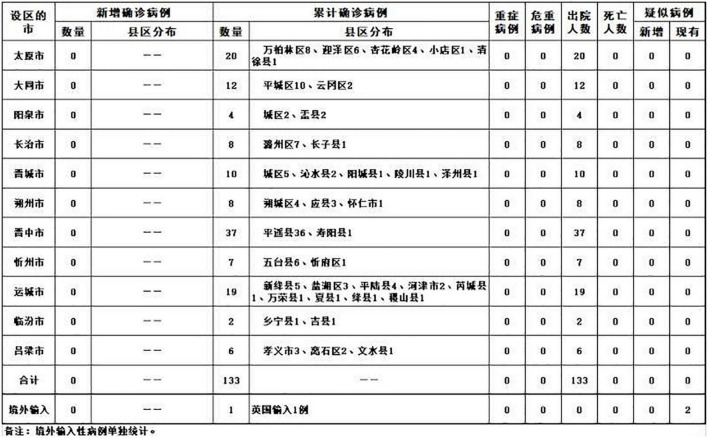
Example of an outbreak trend map.

Third, approximately 29% of the provinces (*n* = 9) used charts to communicate information about the epidemic. For example, the Shanxi Province communicated information about the epidemic through charts since 13 February and up to 13 May 2020, as shown in Figure 3.

**FIGURE 4:**
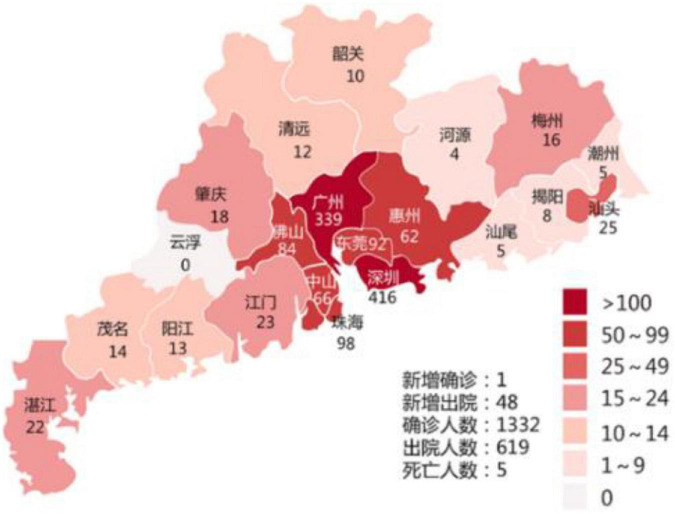
Example of an outbreak chart.

Fourth, 39% of the provinces (*n* = 12) communicated by marking the cases in each district on a local map. For example, the map of the epidemic in Guangdong is shown in Figure 4 (with 20 February 2020, as an example).

**FIGURE 5:**
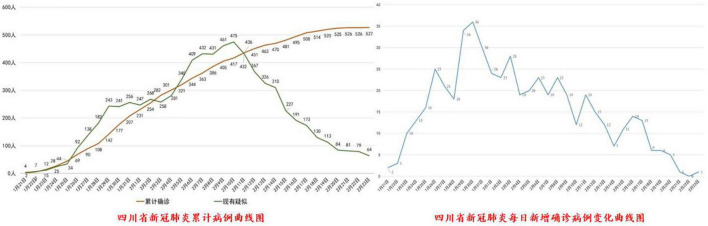
Example of an outbreak map.

Fifth, approximately 32% of the provinces (*n* = 10) have presented the local epidemic development through trend charts. For example, the trend graph of the epidemic in Sichuan (as of 20 February 2020) is shown in Figure 5.

Sixth, approximately 10% of the provinces (*n* = 3) explained and supplemented the situation of the outbreak information by holding press conferences. For example, the Chongqing Health Commission held 72 press conferences as of 12 May 2020.

Seventhly, only one province (Shanghai) converted the text of the outbreak updates into speech through speech synthesis technology, and the public can listen to the outbreak broadcast by clicking on the player attached to the reader.

### 4.3. Selected innovative ways

From the above analysis, it is clear that, in the face of the sudden outbreak of COVID-19, the official agencies represented by the Provincial Health Commissions exhibited different levels of performance in crisis communication. In particular, compared to the communication baseline of the National Health Commissions, each Provincial Health Commission has different degrees of innovation. By coding and analyzing the functional performance of each province based on 17 items, this study summarizes 12 innovative communication ways of the 31 provincial (autonomous regions and municipalities directly under the central government) health commissions in China on outbreak updates as an important crisis communication tool (Table 3).

**TABLE 3:** Categories of innovative ways of updates.

Category	Innovative ways	Description
Content innovation	➀Briefing on individual cases	Publication of the trajectory of confirmed cases
	➁List of risk levels	Publication of dynamically adjusted risk levels on a county-by-county basis
	➂Expert reminder/warm tips	Attached as an expert reminder/tip/risk warning, etc.
	➃Good news bulletin for discharge	Extracting discharge information for a separate broadcast under the heading “Good News!”
	➄Briefing on donations/materials	Disclosure of receipt and use of funds/materials for prevention and control
Form innovation	➅Multiple updates in one day	Briefings were distributed more than once a day
	➆Multilingual briefing	Information on the outbreak is communicated in eight languages, including Chinese, English, French, Germany, Japanese, and Korean.
	➇Synthetic voice broadcasting	Automatic synthesis of text messages of outbreak updates into audio messages
	➈Data list display	Presenting epidemic data by municipality/district in tabular form
	➉Epidemic map	Presenting the epidemic situation by city/district as a heat map
	⑪Trend analysis chart	Visualization of numbers into graphs/bar charts/pie charts etc.
	⑫Press conference	Release information on the outbreak through press conferences

## 5. Coupling analysis

The COVID-19 epidemic is a great disruption to the “psychological order” of the public. Crisis communication for massive psychological intervention is important to restore psychosocial order. Based on the 12 innovative communication ways listed in Table 3, the psychological connotation of the innovative ways was analyzed based on three basic crisis intervention models and the coupling questions were raised to realize the coupling of the ways and psychological intervention (Table 4). The present study’s coupling analysis was also based on the principle of “coupling first, effect second.” This is due to the three factors listed in Table 4. First, previous research studies lack a coupling framework, and coupling mechanisms must be discovered first to lay the groundwork for empirical research of their effectiveness and ways for improvement. Second, this study emphasizes the possibility and potential of large-scale psychological interventions. The universality of the intervention object will come at the expense of effectiveness on specific targets, but this trade-off does not diminish the significance of the large-scale effect. Third, this study intended to offer a fresh perspective on the modernization of emergency management that “care” shall be a sign of progress in crisis communication.

**TABLE 4:** Categories of innovative ways of updates.

Models	Factors	Coupling	
		Content	Form
Equilibrium model	Emotion	➃Good news bulletin for discharge	Null
Cognitive model	Knowledge	➂Expert reminder/warm tips	⑪Trend analysis chart
	Fact	➀Briefing on individual cases ➁List of risk levels	➆Multiple updates in 1 day ➈Data list display ➉Epidemic map ⑫Press conference
Psychosocial transition model	Internal	➂Expert reminder/warm tips	➇Synthetic voice broadcasting
	External	➄Briefing on donations/materials	➅Multilingual briefing

In terms of the equilibrium model, individuals facing COVID-19 and lockdown measures are easily caught in a state of psychological imbalance. The uncertainty and unknowns make it difficult for them to meet their current needs through their original coping mechanisms and methods. The positive content of “good news from the hospital” alleviates individuals’ fears and helps balance public anxiety.

In terms of the cognitive model, the quality of information on the Internet is mixed, confusing the public, and misleading individuals’ judgment of events. The knowledge output of professionals, the pluralistic presentation of scientific data, and the timely update of factual information are all conducive to helping individuals recognize the irrational components of cognition, regain the rational components of thinking, and grasp knowledge and facts in a comprehensive manner.

In terms of the psychosocial transition model, internally, synthetic voice broadcasting helps the blind community to improve their ability to cope with crises. Externally, people in society cannot exist as independent individuals without the influence of the external environment. The public can recover in a caring social environment by grasping information about resource support such as donations or material situations. Multilingual briefing facilitates international collaboration and the integration of global social resources.

## 6. Discussion and conclusion

By identifying the innovative ways of outbreak updates as an important crisis communication tool from 31 provinces (autonomous regions and municipalities directly under the central government) in China and coupling them with crisis psychological intervention models, this study attempted to select innovative ways that have preservation and institutionalization value and propose a better strategy for post-COVID crisis communication and intervention.

(a) Paying attention to the multiple roles of crisis communication at the government, society, and individual levels and making reasonable institutional coupling arrangements. The success of crisis management depends largely on the smoothness of crisis communication. Furthermore, the key to the success or failure of crisis communication lies in whether a sound and complete crisis communication system is established. Crisis communication occupies a substantial proportion of the study of crisis management. However, the importance of crisis communication and its role as an intervention should be reflected in institutional mechanisms for crisis management. We suggest building or strengthening the role of the Publicity or Communication Center of the Health Commission, ensuring the smooth transition between regular communication and crisis communication in the working mechanism, continuously conducting crisis communication rehearsals and capacity accumulation during the normal period, setting up a communication officer position, and recruiting someone who has better departmental coordination ability and learning and innovation ability.

(b) Taking care of psychology and emotions. To be specific, combining rational and emotional narratives in the discourse system. A crisis is an aggregation of facts and feelings. Fear, anxiety, compulsion, tension, and other undesirable social mindsets may intensify the crisis, which needs to focus on the unity of authoritative release and emotional concern in crisis communication. Most of the 31 provinces in China (autonomous regions and municipalities directly under the central government) include tips and warm reminders in their outbreak updates and health tips about special groups such as children, pregnant women, and the elderly. Shanghai also helps those with dyslexia access information through speech synthesis, which can also be seen as an emotional expression of digital humanity. The Japanese poem *“The mountains and rivers are different; the wind and the moon are the same”* printed on the boxes of donated goods was well-received on the Internet in China. In addition to the good effect of macro crisis communication, the micro emotional narrative and care for special groups are essential signs of the modernization of the governance system and capacity.

(c) Developing a crisis communication toolkit to enhance its role as crisis intervention. National, provincial, municipal, and district health commissions represent different levels of crisis communication capacity. Developing a crisis communication toolkit can achieve “twice the result with half the effort” and improve the overall level of communication capacity. By absorbing international practical experience and selecting outstanding innovations presented in this study, a toolkit that serves as a crisis communication infrastructure can be developed to empower crisis management teams at all levels. Toolkit development needs to be based on the principles of uniformity and flexibility. For example, strengthening the uniformity of baseline texts in the case of outbreak updates will ensure the quality of overall crisis communication. Moreover, it will also facilitate global coordination and data mining for social research. Flexibility is about bringing into play the initiative of each crisis management team and emphasizing the inspirational role of innovative cases. From time to time, outstanding crisis communication innovations from home and abroad can be selected and added to upgrade the toolkit.

(d) Institutionalizing multilingual updates and improving global empathy to combat the epidemic. The COVID-19 outbreak has once again proved that we are in a “global risk society” and that humanity is a community of shared destiny. The response to global challenges requires concerted efforts, solidarity, and cooperation by the international community. Language is a significant barrier to international collaboration. Multilingual updates can reduce the cost of international communication, improve international cooperation to combat the epidemic, and make risk monitoring and assessment more effective. A Canadian global infectious disease risk monitoring company collects and processes texts such as official public health agency statements in more than 65 languages to identify the risk of infectious diseases globally at an earlier stage (Bowles, 2020). As machine translation technology improves, the cost of multilingual updates will also continue to decline. The institutionalization of multilingual updates is a concrete manifestation of our open, transparent, and responsible attitude.

(e) Strengthening crisis management training and improving the hard and soft crisis communication skills of crisis management teams. The study finds that localities show different levels of management of epidemic communication and reflect local governments’ governance and innovation capacity. While some provinces are more aware and capable of crisis communication and can innovate on their own, using a combination of innovative ways of outbreak updates, including digital visualization, nearly a quarter of provinces have only a single way of communication. This requires strengthening crisis communication training for crisis management teams at all levels and providing guidance on using the abovementioned crisis communication toolkit to improve crisis communication awareness, enrich crisis communication, and optimize crisis communication effectiveness as a psychological intervention.

## Data availability statement

The original contributions presented in this study are included in this article/supplementary material, further inquiries can be directed to the corresponding author.

## Author contributions

SZ contributed to the conception, data analysis, and manuscript writing of the study. WY contributed to parts of literature obtaining and manuscript writing. XT performed the psychological coupling and analysis with constructive discussions of the study. XL contributed to parts of the conception and coding. All authors contributed to the article and approved the submitted version.
